# Epigenetically-Inherited Centromere and Neocentromere DNA Replicates Earliest in S-Phase

**DOI:** 10.1371/journal.pgen.1001068

**Published:** 2010-08-19

**Authors:** Amnon Koren, Hung-Ji Tsai, Itay Tirosh, Laura S. Burrack, Naama Barkai, Judith Berman

**Affiliations:** 1 Department of Genetics, Cell Biology, and Development, University of Minnesota, Minneapolis, Minnesota, United States of America; 2 Department of Molecular Genetics, The Weizmann Institute of Science, Rehovot, Israel; The University of North Carolina at Chapel Hill, United States of America

## Abstract

Eukaryotic centromeres are maintained at specific chromosomal sites over many generations. In the budding yeast *Saccharomyces cerevisiae*, centromeres are genetic elements defined by a DNA sequence that is both necessary and sufficient for function; whereas, in most other eukaryotes, centromeres are maintained by poorly characterized epigenetic mechanisms in which DNA has a less definitive role. Here we use the pathogenic yeast *Candida albicans* as a model organism to study the DNA replication properties of centromeric DNA. By determining the genome-wide replication timing program of the *C. albicans* genome, we discovered that each centromere is associated with a replication origin that is the first to fire on its respective chromosome. Importantly, epigenetic formation of new ectopic centromeres (neocentromeres) was accompanied by shifts in replication timing, such that a neocentromere became the first to replicate and became associated with origin recognition complex (ORC) components. Furthermore, changing the level of the centromere-specific histone H3 isoform led to a concomitant change in levels of ORC association with centromere regions, further supporting the idea that centromere proteins determine origin activity. Finally, analysis of centromere-associated DNA revealed a replication-dependent sequence pattern characteristic of constitutively active replication origins. This strand-biased pattern is conserved, together with centromere position, among related strains and species, in a manner independent of primary DNA sequence. Thus, inheritance of centromere position is correlated with a constitutively active origin of replication that fires at a distinct early time. We suggest a model in which the distinct timing of DNA replication serves as an epigenetic mechanism for the inheritance of centromere position.

## Author Summary

Centromeres form at the same chromosomal position from generation to generation, yet in most species this inheritance occurs in a DNA sequence–independent manner that is not well understood. Here, we determine the timing of DNA replication across the genome of the human fungal pathogen *Candida albicans* and find that centromeric DNA is the first locus to replicate on each chromosome. Furthemore, this unique replication timing may be important for centromere inheritance, based on several observations. First, DNA sequence patterns at centromeres indicate that, despite high levels of primary sequence divergence, the region has served as a replication origin for millions of years; second, formation of a neocentromere (a new centromere formed at an ectopic locus following deletion of the native centromere DNA) results in the establishment of a new, early-firing origin of replication; and third, a centromere-specific protein, Cse4p, recruits origin replication complex proteins in a concentration-dependent manner. Thus, centromere position is inherited by an epigenetic mechanism that appears to be defined by a distinctively early firing DNA replication origin.

## Introduction

Centromeres are essential components of eukaryotic chromosomes required for proper chromosome segregation to daughter cells. Lack of a functional centromere, or the presence of multiple centromeres, renders chromosomes unstable and prone to mis-segregation and breakage. This genome instability is associated with carcinogenesis and can also result in cell death. An intriguing property of most eukaryotic centromeres that remains poorly explained is their mode of inheritance. In principle, the functional identity of a single locus on a chromosome, such as a centromere, requires that locus to have at least one unique property that distinguishes it from other loci on that chromosome. While a primary DNA consensus sequence would be sufficient to define a single locus per chromosome, most eukaryotic centromeres are not defined at the DNA sequence level (reviewed in [1]–[5]). Thus, for instance, centromeres on different chromosomes in any one species do not share primary DNA sequence between them; furthermore, centromeric DNA sequence diverges between closely-related species while centromeric loci remain syntenic; in rare cases, centromere proteins move to new loci that do not normally function as centromeres. These neocentromeres, which remain stable at their new ectopic loci, have been observed in humans as well as in several model organisms [6]–[8].

Rather than a specific DNA sequence, a unique, conserved histone H3 variant, termed CENP-A/CenH3 (Cse4 in yeasts), distinguishes eukaryotic centromeres from the rest of the chromosome and has inspired the majority of current models of epigenetic centromere inheritance. These models propose that centromeric chromatin structure contains the information necessary to form and maintain centromeres at a given locus. One model suggests that CENP-A and histone H3 are expressed and/or deposited at different times during the cell cycle [9]–[12]. Consistent with this, *S. pombe* CENP-A expression reaches maximal levels just before the G_1_-S boundary [11] and can be deposited in either G1/early S-phase, or via a different pathway in G2 [12]. A related hypothesis suggests that centromeric DNA might replicate at a distinct time during S-phase, and that this may be coordinated with the timing of CENP-A deposition [13] (reviewed in [14]). However, studies in flies and mammalian cells that utilized microscopy measurements of BrdU incorporation to follow the replication timing of centromeric DNA failed to detect a distinct time of replication at centromeres [9], [15]–[17] and thus such models were abandoned. It is important to note that these experiments may lack the resolution and precision necessary to detect replication events within specific regions of the genome. Replication timing microarrays could provide the necessary precision, however centromeres in most eukaryotes typically span hundreds to thousands of kilobases of highly repetitive DNA and are usually not fully sequenced, thereby obviating such analyses. In contrast, centromeres in the pathogenic yeast *Candida albicans* exhibit all the hallmarks of epigenetically inherited centromeres, yet are short (∼ 3 kb), simple and fully sequenced [7], [18]–[21], making this yeast an attractive model system for the study of centromeric DNA properties.

In this study, we used *C. albicans* to study the replication of centromeric DNA. We found that on all chromosomes, the centromere was associated with a replication origin that fires first, and well before all other origins, on that chromosome. Manipulating Cse4 binding by either deletion of a canonical centromere locus or by placing it under the control of a conditional promoter revealed that centromere determinants attract replication components and specify early DNA replication. In addition, we describe a sequence feature of *C. albicans* centromeres - asymmetrical nucleotide composition - that is indicative of stable replication activity over evolutionary time scales. Using phylogenetic comparison, we provide evidence linking the epigenetic inheritance of centromere position with replication activity. Thus, DNA replication timing can serve as the basis for the inheritance of functional centromeres at specific chromosomal sites, representing a novel mechanism of epigenetic inheritance.

## Results

### Centromeres replicate at a distinct time during S-phase

**Figure 1:**
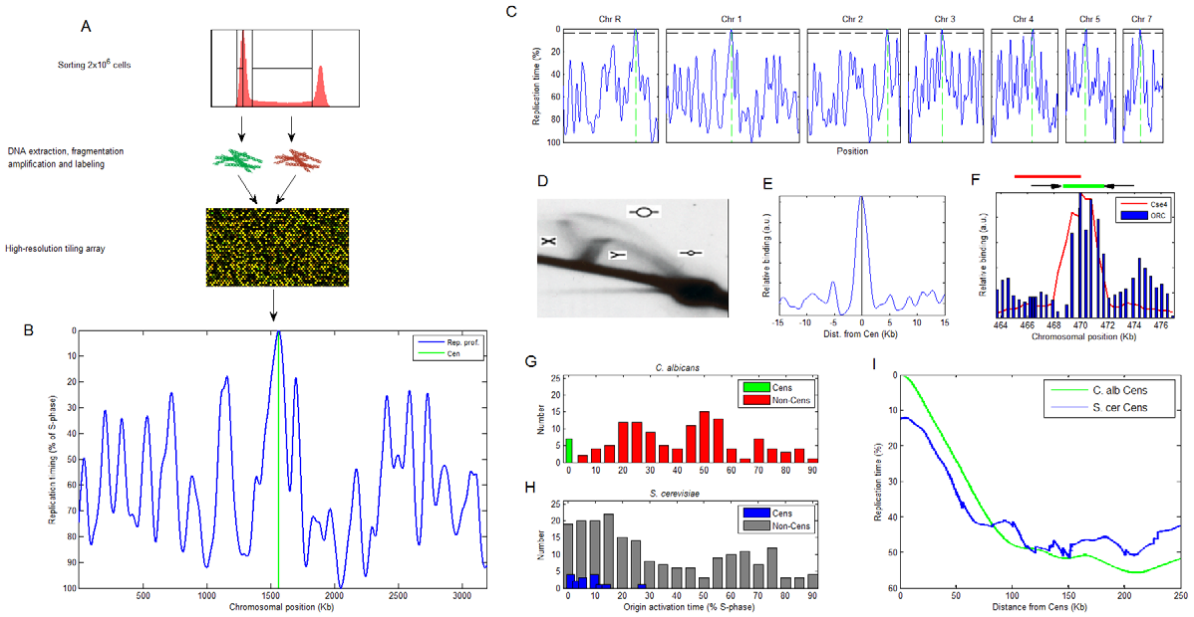
Centromeres are first to replicate in *Candida albicans.* (A) Experimental method for measuring DNA replication timing. The earlier a certain locus replicates, the higher its DNA content will be when averaged over an asynchronous population of S-phase cells. Therefore, measuring copy number along the chromosomes in S-phase cells enables deduction of the genomic DNA replication timing program [22]. (B) The replication profile of chromosome 1. Peaks are predicted origin locations with their height corresponding to activation time. (C) The replication profiles of all chromosomes other than Chr6 (Materials and Methods and Figure S1). Green: centromeres. Horizontal black line: replication timing of the first non-centromeric origin activated in the genome, to denote the clear separation of timing for centromeric versus other origins. (D) 2D gel identifies replication intermediates in *CEN5*. The replication structures represented by each arc are shown [32]. The position of the probe used for the 2D gel is shown in red above (F). (E) ORC ChIP-chip values averaged over all chromosomes, aligned at the GC skew zero-intersection point (see text). See Figure S2 for the individual chromosome data. (F) ChIP of ORC retrieves *CEN5* sequence. The ChIP result for Cse4 [7] is shown for comparison. The location of the centromere (green line) and flanking inverted repeats (black arrows) are shown above. (G,H) Distribution of relative activation times for centromeric and non-centromeric replication origins in (G) *C. albicans* and (H) *S. cerevisiae* (data from [22]). (I) Centromere position effect.

We determined the genome-wide DNA replication timing profile at high temporal and spatial resolution for all eight *Candida albicans* chromosomes (Figure 1A). Briefly, asynchronous log phase cells were sorted by fluorescence activated cell sorting into G1 and S phase fractions. DNA from 2×10^6^ cells from each fraction was extracted, differentially labeled with fluorescent dyes and hybridized to genomic tiling arrays. The data were smoothed and replication timing was displayed as a function of chromosome position. In these replication profiles, peaks represent replication origins and the height of each peak reflects the relative timing and/or efficiency of replication from that origin. When applied to *S. cerevisiae*, this method identifies known replication origins at a resolution of ∼5 Kb ([22]; Materials and Methods).

The replication profiles exhibited a striking feature: centromeres were always proximal to a predicted replication origin and remarkably, these centromeric origins were the first to fire on each chromosome. Furthermore, the activation timing of these centromere-associated origins was clearly separate from the activation time of non-centromeric origins (Figure 1B and 1C and Figure S1). The colocalization of replication origins with centromere position is further supported by several lines of evidence. First, two-dimensional DNA gels detected a replication bubble structure, indicative of an active origin, at the chromosome 5 centromere DNA region (*CEN5*) (Figure 1D). Second, genome-wide chromatin immunoprecipitation (ChIP-chip) experiments of the Origin Recognition Complex (ORC), that is essential for replication initiation, revealed the presence of ORC within the centromere regions of each of the eight chromosomes (Figure 1E and Figure S2). These ORC binding sites were among the strongest in the genome (Figure S2 and data not shown). Third, ChIP followed by quantitative PCR of *CEN5* detected ORC localization within *CEN5* in correlation with the position of Cse4 (Figure 1F; [7]). Thus, centromeres in *C. albicans* contain replication origins that are the first to replicate on each chromosome.

The distinct replication timing of the centromeres is manifested in the distribution of origin activation times in the *C. albicans* genome: non-centromeric origins are largely absent from the beginning of S-phase and are otherwise activated throughout S-phase (Figure 1G). In contrast, in *S. cerevisiae* (that has genetically-inherited point centromeres), most origins are activated in the beginning of S-phase, at a time that is not distinct from that of centromere-proximal origins (Figure 1H; [23]). Thus, *C. albicans* achieves genomic replication with significantly fewer early-activated origins relative to *S. cerevisiae*. However, the paucity of early origins does not result in a longer S-phase in *C. albicans* compared to *S. cerevisiae*. This could be explained by a difference in the distribution of active origins with different activation timing along the chromosome: in *S. cerevisiae*, early origins tend to cluster in close proximity, ultimately resulting in ∼2-fold more active origins per DNA length than in the *C. albicans* genome. In *C. albicans*, the spacing of early and late origins is more evenly interspersed (Figure S3). This more regular spacing of origins along the chromosome is presumably more efficient, enabling utilization of fewer origins in *C. albicans*.

Previous work described a replication timing position effect in *S. cerevisiae* [23], in which centromeres are located within a region of several tens of kilobases of early replicating DNA (and see below). A similar, albeit significantly stronger effect is evident in *C. albicans* (Figure 1I) where a region of up to ∼100 kb surrounding the centromere replicates earlier than the genomic average, further emphasizing the distinctiveness of centromere replication timing in *C. albicans*.

### Centromeres are effectors of DNA replication timing

**Figure 2:**
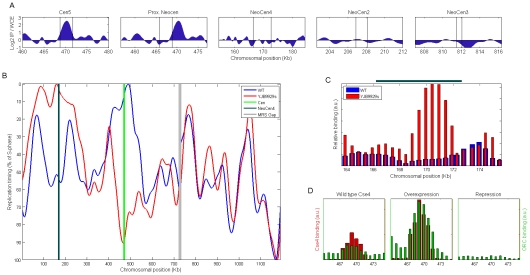
Neocentromere formation causes replication timing advancement. (A) ORC binding (determined by ChIP-chip) in wild-type cells at *CEN5* and at chromosome 5 loci with the potential to form a neocentromere [7]. ORC clearly binds at *CEN5* and is not evident within the loci that have been shown to form neocentromeres following deletion of *CEN5*. Regions identified as centromeric or capable of forming neocentromeres (by virtue of Cse4 binding in the corresponding *cen5Δ* strains) are located between the two vertical lines. (B) The replication profile of chromosome 5 in wild-type and in strain YJB9929s, in which *CEN5* was deleted and a neocentromere formed on the left chromosome arm [7]. MRS: Major Repeat Sequence. (C) ChIP reveals an ORC binding site within the neocentromere region (dark green line above the plot; [7]) in strain YJB9929s that is not present in the wild-type strain. We note that ORC binding provides a more precise indication of the actual location of an origin than the replication timing profiles; thus, the offset of the replication peak in (B) from the neocentromere site is likely explained by experimental noise. (D) Overexpression or repression of *CSE4* (Materials and Methods) is accompanied by consistent changes in ORC binding strength at *CEN5*.

The colocalization of centromeres and early replication origins could be caused by replication origins recruiting centromeric determinants. Alternatively, centromeres could recruit replication determinants. To address the direction of dependency between centromeres and replication origins, we exploited *C. albicans* strains in which the native chromosome 5 centromere had been deleted and a heritable neocentromere had formed at a different novel locus on the chromosome in each strain [7]. These strains provide a unique opportunity to address the order of this dependency. In four neocentromere strains, the centromere shifted to non-telomeric loci that were not ORC-binding sites in the wild-type progenitor strain (Figure 2A and 2C). This indicates that neocentromeres do not form at pre-existing replication origins.

We then assayed replication timing in a strain that acquired a stable homozygous neocentromere following deletion of *CEN5* DNA and homozygosis of the entire chromosome (neoCEN4, strain YJB9929s; see Materials and Methods; [7]). Origin activity at the *CEN5* region was completely lost, indicating that origin determinants had resided within the deleted centromeric DNA (Figure 2B). Strikingly, neocentromere formation was associated with the appearance, in the neoCEN4 region, of a new replication origin that was the first to replicate in the chromosome (Figure 2B). Consistent with the idea that a *de novo* origin formed at neoCEN4, a new ORC binding site in the neoCEN4 region was readily evident by ChIP-PCR only in the strain that had formed a neoCEN and not in its wild-type parental strain (Figure 2C). Of note, the replication timing of origins flanking the centromere and neocentromere was also altered, possibly reflecting a broad position effect exerted by centromeres on the replication timing of flanking origins of replication. This resembles the position effect that mediates generally earlier replication timing within tens of kilobases surrounding conventional centromeres (Figure 1I). The profiles of chromosomes 2 and 3, which would not be expected to be altered by deletion of *CEN5*, were indeed similar between the neocentromeric and wild type strains (correlation between the profiles r = 0.92−0.95 versus 0.36 for chromosome 5).

To further test the hypothesis that centromeric activity can recruit replication determinants, we used a conditional promoter to manipulate the level of Cse4 expression and assayed the effect of Cse4 levels on the level of ORC-binding at centromeres. Consistent with the idea that centromeres recruit origins of replication, we found that over-expression of Cse4 resulted in increased levels of ORC at *CEN5*, while repression of Cse4 expression resulted in reduced levels of ORC (Figure 2D). Taken together, we conclude that centromeric determinants confer both the presence of replication origins and a distinct, early replication timing to the loci at which they reside.

### Sequence asymmetry at centromeric DNA suggests constitutive origin activity correlated with epigenetic inheritance of centromere positions

**Figure 3:**
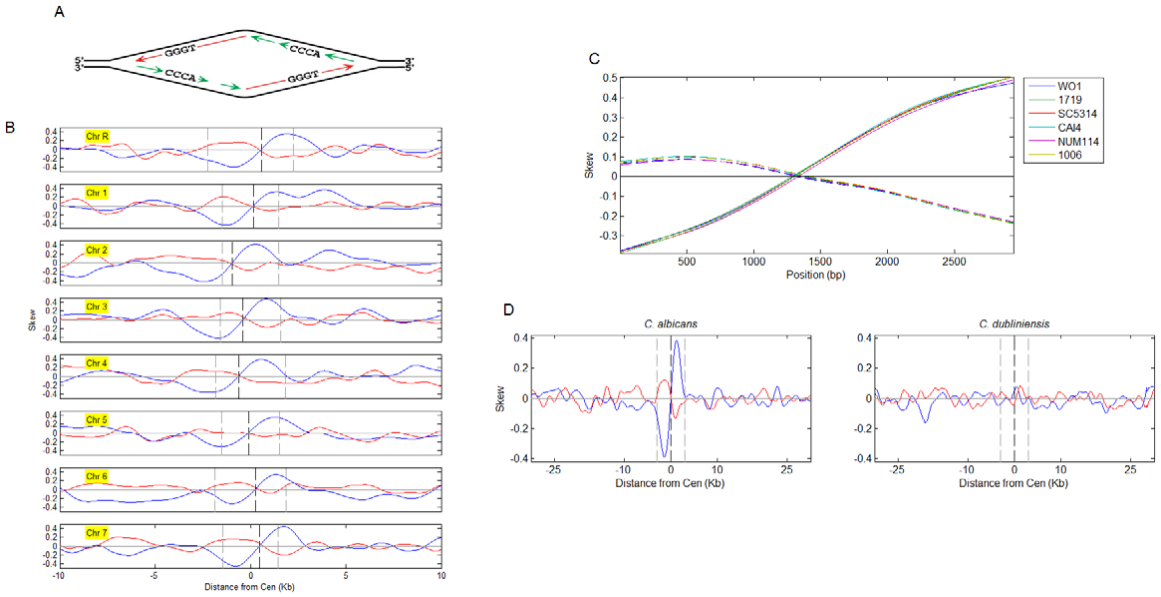
Nucleotide skew patterns and their correlation to syntenic conservation of centromere position. (A) A diagram of GC skew formation. G, and to a lesser extent, T nucleotides, are enriched on the leading strand. The location of the replication origin is the point of replication strand switch as well as the point of GC and AT skew orientation reversal. (B) GC (blue) and AT (red) skew at the *C. albicans* centromere regions. The opposite orientation of the GC and AT skews depends on the way the skews are defined (i.e. the same analysis with relation to ‘TA’ instead of ‘AT’ skews would result in the same orientation). Skew curves represent the degree to which base-pairs are oriented with a particular base on one (value of 1) or another (−1) of the strands (0- no skew). Centromeres are aligned at the center of the published centromere borders (dashed grey lines). Dashed black lines: GC skew zero-intersection points. GC-skewed regions also exhibited high GC content (Figures S4, S5, S6, S7); GC content at the centromeres exhibited a local drop within a region of increased GC content; nucleosome occupancy appeared to follow a similar pattern (Figure S6). (C) GC skew (solid lines) and AT skew (dashed lines) for *CEN1* of the *C. albicans* strains indicated. Based on sequence data for a DNA fragment containing *CEN1* from Ref. [20] (x-axis is position on the sequenced fragment). For strain WO-1, where the complete genome sequence is available, skew patterns were conserved at all centromeres (data not shown). (D) Average GC and AT skew patterns for *C. albicans* (based on individual chromosomes shown in (A) and *C. dubliniensis* (individual chromsomes are shown in Figure S9). Chromosomes were aligned at the GC skew zero-intersection points (dashed black line) and skew signals were averaged. Dashed grey lines delimit 3 kb regions to each side.

As in higher eukaryotes, *C. albicans* has regional centromeres with different primary sequences at each chromosome [18], [20]. Furthermore, centromeric DNA sequences are highly divergent between strains and related species, mutating at rates higher than those at intergenic regions and synonymous sites [21]. Despite this lack of homology, sequence analysis revealed a common feature among *C. albicans* centromeres: all of them have a sequence pattern that has been detected previously at replication origin sites of hundreds of bacterial, archeal, viral and organellar genomes that have a single replication origin in their genome [24]. This pattern, indicative of constitutive replication origin activity, is an asymmetric GC skew, in which G nucleotides are more abundant than C nucleotides on one side of the centromeric replication origins, and G nucleotides are less abundant than C nucleotides on the same strand on the other side of the centromere (Figure 3A, 3B, 3D). For A versus T nucleotides, a similar skew pattern was observed, in the opposite direction relative to the GC skew, with a lower magnitude, and with essentially the same position of switch in skew direction. A similar AT skew pattern is present in many of the species with single-origin genomes.

Such skew patterns are inferred to be a consequence of mutations that accumulate in a strand-specific manner: specific substitutions occur at different rates on the leading and lagging strands and the leading and lagging strands switch identities at the point of replication initiation (Figure 3A). Thus, a nucleotide skew pattern implies the presence of a replication origin at the point of symmetry switch [24]. This is consistent with our results detecting the presence of replication origins within centromeric regions. In particular, strong ORC-binding sites reside in very close proximity to the points of skew sign switch (Figure 1E and Figure S2)).

Moreover, skew patterns accumulate over evolutionary time scales, only in cases where a replication origin is consistently active for many generations; hence skews are evident in most genomes with single origins. Conversely, and despite the observation that replication fork asymmetry also causes a strand bias in mutagenesis experiments [25] in *S. cerevisiae*, skew signals are rarely detected in eukaryotic genomes, presumably because eukaryotic replication origins are not constitutively active at specific loci over long time periods.

Thus, the identification of asymmetrical skew patterns at centromeric replication origins of *C. albicans* suggests that these origins have been active in most, or all, cell cycles for many generations. Indeed, skew patterns are conserved among *C. albicans* strains that represent divergence times of 1-3 million years (Figure 3C; [20]). Furthermore, skew patterns are virtually identical at all centromeres in *C. albicans* and *C. dubliniensis* (Figure 3D and Figure S9), which diverged from each other ∼20 million years ago and share complete conservation of centromere synteny, yet no conservation of centromere primary DNA sequence [21]. The conservation of skew patterns at centromeres in both organisms suggests that DNA replication activity is intimately associated with the mechanism that has ensured the epigenetic inheritance of centromere synteny.

In scanning the entire *C. albicans* genome sequence, we found that the strongest skew patterns were at centromeres, but that skew patterns were also identifiable at many telomeres (Figures S4, S5, S6, S7). No skew patterns different than background were seen in any of the loci where neocentromeres have been observed to form (data not shown), indicating that skewed DNA is not an attractor for centromeric proteins. Rather, the lack of skew patterns at neocentromeric loci is consistent with the idea that skews only arise following constitutive origin activity at centromeres over evolutionary time scales.

### Evolutionary conservation of the centromere-early origin association

A DNA sequence pattern characterizing epigenetically-inherited centromeres provides a potential tool for predicting centromere locations in other species. Indeed, we found one distinctive skew pattern per chromosome, providing a prediction of centromere (and origin) locations, in two additional members of the *Candida* clade, *L. elongisporus* and *C. lusitiniae*, as well as in the more distantly related yeast, *Yarrowia lipolytica* (Figures S8, S9; Table S1). In *Y. lipolytica*, our approach re-identified the five known centromere locations (and predicts the centromere location of the sixth chromosome) in this species. Remarkably, *Y. lipolytica* centromeres were identified by searching for chromosomal sequences that function as DNA replication origins and subsequent work showed that, for *Y. lipolytica* plasmids, replication origin and centromere activity are inter-dependent [26], [27].

**Figure 4:**
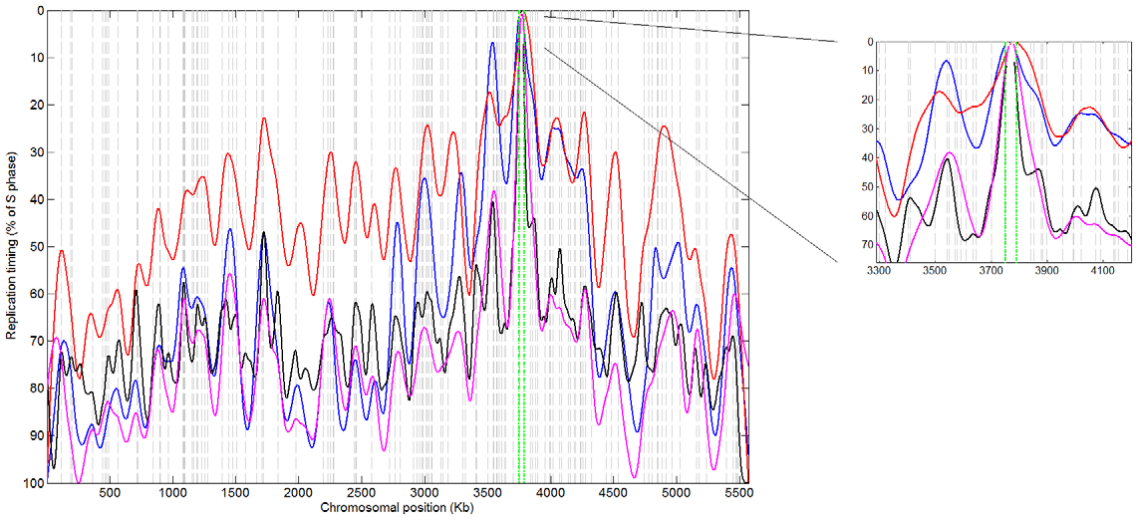
Replication timing profile of *S. pombe* chromosome 1. Replication timing microarray data for chromosome 1 from [35] (blue), [34] (red- time course experiment; black- HU experiment), and [36] (magenta) show early replication at *CEN1*. Green lines: centromere location; dashed grey lines: ORC binding sites that also incorporated BrdU [38].

*S. pombe* centromeres also include several replication origins that fire very early in S-phase despite being embedded within heterochromatin [28]. We re-analyzed *S. pombe* replication timing microarray data (Materials and Methods), and found that *CEN1*, the only centromere that could be probed at sufficient resolution, is the first locus to replicate on chromosome 1 (Figure 4). Notably, no skew patterns were observed at *S. pombe* centromeric regions (data not shown). This is likely due to the presence of more than one origin within these regions. We suggest that centromeric skew patterns may be unique to species with smaller regional centromeres. In contrast, in a clade containing the *Saccharomyces* species (see Figure S9A), genetic, point-centromere positions have become fixed in DNA sequence [29] and do not coincide directly with replication origins. Nonetheless, generally early DNA replication surrounding centromeres (Figure 1H and 1I) could be an evolutionary remnant of replication-timing-dependent, epigenetically-inherited centromeres. Taken together, the association between centromeres and replication origins is conserved at least across a wide range of yeast phylogeny (Figure S9A). Since neither DNA sequence nor sufficient replication timing data are available for centromeric regions of higher model organisms, it remains to be determined whether this association is common among other eukaryotic species.

## Discussion

**Figure 5:**
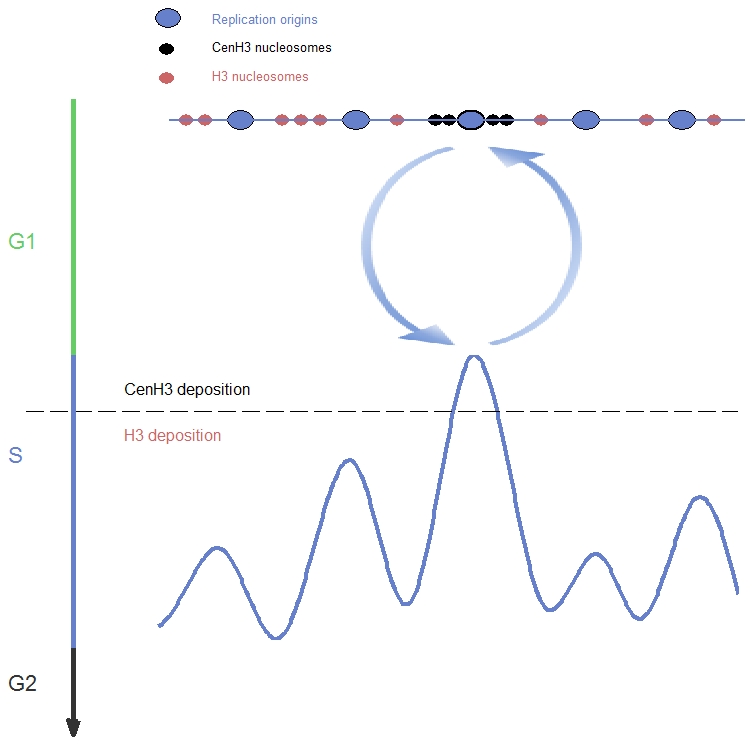
A model of DNA replication-timing–dependent epigenetic centromere inheritance. Two processes contribute to a self-reinforcing feedback loop: replication origin sites embedded within centromeric chromatin (upper part) are activated earlier than other origins (lower part); and assembly of centromeric chromatin occurs in very early S-phase, while canonical chromatin is replicated later.

This study provides the first comprehensive analysis of DNA replication of epigenetically-inherited centromeres. Our results show that centromeres replicate at a distinct time from the rest of the chromosome, centromeric determinants can change the replication time of the loci at which they reside, and the DNA replication properties of centromeres are linked to their epigenetic inheritance over evolutionary time scales. Based on this, we propose a self-reinforcing, positive-feedback loop model, in which centromeric determinants affect DNA replication timing and in turn, a distinct replication time facilitates the recruitment of centromeric determinants to that specific locus (Figure 5). Specifically, centromere site specification by Cse4 nucleosomes recruits factors that mediate replication initiation at a distinct time at the very beginning of S-phase (Figure 2). Furthermore, we speculate that early, distinctive replication timing, in turn enables subsequent deposition of centromere-specific proteins such as Cse4 nucleosomes at the region that is first to replicate, for instance due to elevated levels of these proteins in the very beginning of S-phase [11]–[14]. In particular, it has previously been shown in *S. pombe* that expression of Cse4 occurs from late-M to G1/S phase, and precedes maximal expression of histone H3 in S-phase [11]. Our calculations suggest that centromeric chromatin is replicated very rapidly- within the first <20 seconds of S-phase in *C. albicans* (Materials and Methods). Further studies will be required to link the distinct timing of CENP-A deposition and the time of centromere replication.

This self-reinforcing association between centromere determinants and early replication timing does not require a particular underlying DNA sequence, establishing it as an epigenetic system. This epigenetic system determines centromere position, as well as replication timing and the constitutive nature of centromeric replication origins, which our results show for the first time can be determined epigenetically. Early and constitutive replication are directly related to each other, as a locus that replicates first will never be passively replicated and hence will be active in every cell cycle. The combination of constitutive origin activity and the relaxation of sequence constraints that stems from the epigenetic nature of this system, enables the underlying DNA to mutate at elevated rates, explaining previous observations that centromeric DNA mutates at high rates while centromere position remains unaltered [21], [30]. We now show that centromeric DNA in several yeast species mutates with a specific sequence bias due to the influence of a centromere-associated replication origin.

We describe a novel type of epigenetic inheritance mechanism that is directly related to the fundamental mechanism of genetic inheritance in that both depend on DNA replication. Centromeres are maintained at specific chromosomal positions in a highly robust manner that is crucial for ensuring high-fidelity chromosome segregation; thus, this replication-timing-dependent epigenetic inheritance mechanism may confer a level of stability unprecedented for an epigenetic system. Finally, another attractive aspect of this model is that, instead of requiring a specialized mechanism for inheritance of centromeric nucleosomes outside of S-phase [11], [17], it directly connects centromeric nucleosome inheritance with DNA replication, similar to the inheritance of canonical nucleosomes that occurs following passage of the replication fork.

## Materials and Methods

### Strains and media

Strains SC5314 (wild type; diploid) and YJB9929s [7] (homozygosed for chromosome 5 subsequent to neocentromere formation) were grown in YPAD media at 30°C. Strain CAKS3b in which Cse4 expression is under the control of the glucose-repressible *PCK1* promoter [31] was grown in YPA-succinate to induce Cse4 overexpression and diluted into fresh YPA-succinate or into YPAD to repress expression and grown for 6 hours at 30°C before harvesting cells.

### Replication timing

FACS and replication timing microarray experiments and analysis were as previously described [22]. Agilent arrays (2×105 format) were custom designed with probes spaced every 140 bp, on average, across the entire genome (assembly21). The experiment was repeated four times with correlation and autocorrelation values comparable to those previously obtained [22] (data not shown), validating the high quality of the data. Strain YJB9929s was repeated twice with a microarray that included the same probes for chromosomes 2, 3 and 5 only. Microarray data have been submitted to GEO (http://www.ncbi.nlm.nih.gov/geo/) with accession number GSE17963.

The experimental repeats were weighted-averaged using Tukey's biweight method [22] and the averaged data was smoothed using a smoothing spline (as implicated in the Matlab function Csaps), which optimizes the degree of curvature versus deviance from the data, with the parameter chosen determining the weight of these two criteria. The parameters chosen for the different chromosomes were as follows (numbers are the -log10 of the parameters provided to Csaps): ChrR: 16; Chr1,2: 15.75; Chr3: 15.25; Chr4; 15; Chr5,6,7: 14.75 (generally a function of chromosome length). This choice of parameters maximized the similarity to the *S. cerevisiae* data with respect to the significance of frequency components retained (data not shown), enabling valid comparison of the number of replication origins between *S. cerevisiae* and *C. albicans* (Figure S3).

The rDNA and chromosome 5 Major Repeat Sequence (MRS; which is larger than 50 Kb on chromosome 5) loci were treated as data gaps; the corresponding chromosomes were smoothed separately on each side of these gaps (with the same parameter). Scaling the entire genome, rather than individual chromosomes, to 0–100, had little if any effect on the data.

### Origin analysis

2D gels were performed as previously described [32]. Chromatin immunoprecipitation was as previously described [7] with polyclonal antibodies against the entire ORC complex [33] kindly provided by Stephen P. Bell. PCR was performed with 33 primer pairs interrogating the *CEN5* region or 24 primer pairs for the neocentromere region; data was averaged over two experimental repetitions and three consecutive primer pairs. RT-PCR was performed in duplicates using the LightCycler 480 system according to the manufacturer's instructions. ChIP-chip was performed in nine biological repeats according to Agilent protocols and will be described in detail elsewhere together with a complete list of replication origin sites (A.K., H-J.T, L.B. and J.B, manuscript in preparation). Results remained unaltered when normalizing to probe GC content.

### Analysis of *S. pombe* replication microarray data

Datasets used were: a 65, 75, 85, 95 minutes S-phase time course experiment [34]; a replication timing in HU experiment performed in three repeats[34]; a single experiment using ssDNA mapping [35]; and a two time point (2 h, 4 h) S-phase time course experiment [36]. Chromosomes 2 and 3 had probe gaps of ∼50–80 Kb in the centromere region and thus were not amenable to centromere replication timing analysis. Data from each dataset was weighted-averaged over three consecutive probes and all the time points/repetitions of that experiment and smoothed using spline parameters (as above) of 17 for all experiments and 16 for the HU experiment (use of a parameter of 17 did not alter the result for the centromere). This data analysis approach effectively removes outlier data points that prevented the previous identification of *CEN1* as the first locus to replicate on chromosome 1.

### Time to replicate centromeric DNA

To avoid influences of the data smoothing on replication fork progression near origins [22], we linearly interpolated the replication profiles from peaks to valleys. Replication timing was converted to minutes by multiplying replication time in percent by the total duration of S-phase in minutes (Figure 1). The latest replication timing of the loci 3 Kb from each side of the centromere-proximal origin on each chromosome (excluding chromosome 6 as indicated above) was the time to replicate each centromere.

### Sequences and gene annotations

Genome sequences were obtained from the following sources: *Candida albicans* (assembly 21): from CGD (*Candida* Genome Database; http://www.candidagenome.org/); *Saccharomyces cerevisiae*: from SGD (*Saccharomyces* Genome Database; http://www.yeastgenome.org/); *Candida dubliniensis, Schizosaccharomyces pombe*: from The Wellcome Trust Sanger Institute (http://www.sanger.ac.uk/sequencing/Candida/dubliniensis/), with gene annotation from GeneDB (http://www.genedb.org/genedb/); *Candida albicans* strain WO-1, *Candida guilliermondii*, *Candida lusitaniae*, *Candida parapsilosis*, *Candida tropicalis*, *Debaryomyces hansenii*, *Lodderomyces elongisporus*: from the Broad Institute (http://www.broadinstitute.org/annotation/genome/candida_group/MultiHomehtml); *Pichia stipitis*: from JGI (http://genomejgi-psf.org/Picst3/Picst3homehtml); *Yarrowia lipolytica*: from Genolevures (http://www.genolevures.org/indexhtml) (with centromere positions from [37]).

### Sequence skew analysis

GC skew was calculated as (G−C)/(G+C), AT skew as (A−T)/(A+T), and each was smoothed using a smoothing spline with parameters equivalent to smoothing by sliding windows of ∼1.5 Kb. For identifying strong skew patterns in the genome (see Figure S5), we categorized intergenic regions by the combination of length, GC content, and either of several parameters of: GC skew, AT skew, GC content, proximity of the latter to each other (of zero-intersection points for skews and extrema for GC content), and combinations thereof. We then searched for a discrete cluster in any of these parameters.

## Supporting Information

Figure S1 Replication profile of chromosome 6. As in Figure 1B. *CEN6* replicated within the first 1% of S-phase with another origin on chromosome 6 replicating at 0%; however, in the vicinity of both origins there were large (>5 Kb) probe gaps; in addition, *CEN6* is telocentric. For these reasons we consider the centromere replication timing for this chromosome as less reliable. Chromosome 6 was also not included in the *C. albicans* data used for generating Figure 1G and 1I.
(0.14 MB TIF)

Figure S2 ChIP-chip data for all centromeric regions. The green lines correspond to the published centromere borders. The black line is the GC skew zero-intersection point (Figure 3B). x-axis: chromosomal position (Kb). y-axis: normalized log2 IP/WCE.
(0.12 MB TIF)

Figure S3 Properties of the replication program. Distribution of (A) origin initiation time, (B) replication termination time, (C) inter-origin distances, and (D) inter-origin time differences, for *C. albicans* (blue) and *S. cerevisiae* (green). Data from all the chromosomes was used to generate these figures. Origins numbers (and hence inter-origin distances) depend on smoothing parameters, and these were chosen to be maximally consistent between the two species (Methods). Our conclusion that the density of replication origins is significantly lower in *C. albicans* can also be reached independently by autocorrelation analysis, which is applied prior to smoothing (see [22]; data not shown). *S. cerevisiae* has more origins in early S-phase (A) which are clustered in space (C) and time (D) and therefore terminate early (B), thereby not contributing to the overall speed of S-phase. In *C. albicans*, this class of origins is not present, and origins are instead more uniformly distributed in S-phase (A), are more separated in space (C) and time (D) and are associated with replication forks that extend over a larger portion of S-phase, thus terminating late (B). The percentage of *C. albicans* cells in G1, S and G2 phases (Figure 1A) was 47%, 24% and 29%, respectively. This corresponds to 30.5, 15.6 and 18.9 minutes, for a total generation time of 65 minutes. In comparison, S-phase in *S. cerevisiae* lasts 17 minutes [22]. Thus, while length of S-phase is not extended in *C. albicans* relative to *S. cerevisiae*, in *C. albicans* there is less origin redundancy.
(0.19 MB TIF)

Figure S4 Identification of skew locations. Since GC skew is also associated with high GC content, we used both criteria in order to scan for skew patterns. In the figure, each dot represents an intergenic region. Centromeres are represented by open diamonds and telomeres by ‘+’ signs. The Y-axis refers to the summed skew level for GC skews that change from negative to positive (the highest score was used for intergenic regions which had more than one such skew pattern). The colorcode denotes the length of the intergenic region. Besides the eight centromeres, nine telomeric sites could be identified with comparable levels of GC skew and even higher GC content (and see Figure S5). GC skews at telomeres have also been described in *S. cereviaise* [Gierlik et al], however they are single sided rather than asymmetrical (data not shown), probably arising due to the uni-directional replication from the terminal origins on each chromosome to the ends of that chromosome. Two additional telomeric sites (one of them, Tel6R, in a short intergenic region- see Figure S5) and two internal replication origins could be identified with weaker skew levels. The non-centromeric origins associated with skew signals were not early replicating compared to average. Other than those shown in the figure, replication origins in general did not have stronger skew levels than non-origin sites (not shown). The high correlation between strong skew patterns and elevated GC content (see also Figures S6, S7) suggests that the same mutational mechanism may cause both. Centromere and telomere loci could also be identified with other skew parameters, such as a minimal GC skew level on either side of a zero-intersection point associated with a reverse-sign AT skew. [Gierlik A, Kowalczuk M, Mackiewicz P, Dudek MR, Cebrat S (2000) Is there replication-associated mutational pressure in the Saccharomyces cerevisiae genome? J Theor Biol 202: 305–314.]
(0.07 MB TIF)

Figure S5 Telomere skew patterns. As in Figure 3A; left and right *C. albicans* telomeres are shown on the left and right subplot columns, respectively. Dashed grey lines- borders of the intergenic region containing the skew pattern; when one dashed gray line is present, the other is the end of the chromosome. Red boxes denote telomeres identified by the genomic search for GC skew (Figure S4). The DNA region from the most terminal replication origin site to the corresponding end of the chromosome is always replicated in one direction and will thus show skew patterns, depending on the presence of mutational strand biases in the studied organism, and the absence of selective pressure in this region. For instance, GC and AT skews are observed at *S. cerevisiae* telomeres albeit they are not asymmetrical but rather single-sided (data not shown). This is consistent with multiple potential origins sites at telomeres [33] that are active alternatively. The identification of asymmetrical skew patterns at *C. albicans* telomeres suggests that they contain a single, isolated, active replication origin. This is also consistent with the differences in telomere replication timing between the two yeasts (Figure 1H).
(0.15 MB TIF)

Figure S6 GC content and nucleosome occupancy at *C. albicans* centromeres. (A) Averaged GC content for the centromere regions (black; as in Figure 3B) reveals a local drop at the GC skew zero-intersection point. Since ORFs have a higher GC content than intergenic regions (see Figure S7), we also analyzed GC content for noncoding sequences only, i.e. intergenic regions and 4-fold degenerate (synonymous) 3rd codon positions (blue). This revealed the elevated GC content of the centromeres relative to surrounding sequence. (B) Analysis of GC skew for only non-coding sequence provides the same skew patterns as for all sequence (Figure 3D). (C) Averaged nucleosome occupancy (data from [Field, et al. (2009)]) for the centromere regions, aligned at the GC skew zero-intersection point as above. Previous work showed that replication origins reside in regions with low nucleosome occupancy [Field, et al. (2008)], however centromeres have high nucleosome occupancy [Segal, et al.]. The centromeres/origins in *C. albicans* appear to follow both trends, with a local drop in nucleosome occupancy flanked by short regions of high occupancy. This pattern resembles that of the GC content data (A) and was observed only for *in vivo* nucleosone occupancy data and not for *in vitro* data (data not shown). This implies that physiological activity and not DNA sequence *per se* affects nucleosome occupancy in the centromere regions. [Field Y, Fondufe-Mittendorf Y, Moore IK, Mieczkowski P, Kaplan N, et al. (2009) Gene expression divergence in yeast is coupled to evolution of DNA-encoded nucleosome organization. Nature Genetics 41: 438–445. Field Y, Kaplan N, Fondufe-Mittendorf Y, Moore IK, Sharon E, et al. (2008) Distinct modes of regulation by chromatin encoded through nucleosome positioning signals. PLoS Comput Biol 4: e1000216. doi:10.1371/journal.pcbi.1000216. Segal E, Fondufe-Mittendorf Y, Chen L, Thastrom A, Field Y, et al. (2006) A genomic code for nucleosome positioning. Nature 442: 772–778.]
(0.15 MB TIF)

Figure S7 GC content of centromeres. Shown are the distributions of GC content for ORFs (green), intergenic regions (black) and centromeres (red).
(0.16 MB TIF)

Figure S8 Since skew patterns provide a sequence footprint for ancient centromere-origins, we asked if similar patterns could be identified over a broader phylogenetic range by searching several yeast genomes for intergenic regions with distinctive skew and GC content values (Methods and see Figure S4). The figure shows the average skew and GC content patterns, with individual chromosomes shown in Figure S9. Skew and GC content (black) are shown on the same scale, as level or fraction, respectively. We identified skew patterns, which appeared only once per chromosome, in *L. elongisporus* (A) and *C. lusitinae* (B), as well as in the distantly related species *Yarrowia lipolytica* (C). In *Y. lipolytica*, our approach re-identified the five known centromere positions in this organism [26]. Remarkably, the latter were identified by searching for chromosomal sequences that function as DNA replication origins [27] and subsequent work showed that, for *Y. lipolytica* plasmids, replication origin and centromere activity are inter-dependent [26]. Interestingly, an AT skew was present to similar extents in the three species as it was in *C. albicans* and *C. dubliniensis* (Figure 3); in contrast, GC skew followed an evolutionary path from being not present (in *Y. lipolytica*), to equivalent in magnitude to the AT skew (in the predicted centromeres), and to significantly stronger (in *C. albicans* and *C. dubliniensis*). GC content was correlated with the extent of GC skew: it showed a local drop when a GC skew was absent, but was progressively higher with the increase in GC skew levels (see also Figure S7). Together, this suggests that different replication-dependent mutational mechanisms operated in different lineages.
(0.06 MB TIF)

Figure S9 Skew patterns for different species. (A) Phylogenetic tree of species analyzed in this study and related species (modified from Genolevures (http://wwwgenolevuresorg/indexhtml)). WGD: whole genome duplication. ‘CUG’ clade: the *Candida* species clade, in which the CUG codon encodes Serine instead of Threonine. ‘Point centromeres’: centromeres defined by a short consensus sequence. (B–F) As in Figure 3B; GC and AT skews for separate chromosomes in the indicated species. Grey- borders of the intergene containing the skew pattern, except for *C. dubliniensis*- borders of reported Cse4 binding sites. See Table S1 for coordinates of skew zero-intersection points and corresponding intergenic region borders. No skew signals were identified in *C. guillermondii*, *D. hansenii* and *C. tropicalis*. However, since centromere locations are unknown for these species, it remains possible that centromeres are associated with skew and GC content patterns that are not strong enough to be recognized unequivocally. No skew signals were identified in the S. *pombe* genome despite the presence of active (and early) replication origins within the centromere regions [28]; this could be explained by alternate firing of different origins within the centromere region, by a different mutational mechanisms, or by functional constraints. No skew signals were identified in the *S. cerevisiae* genome, with the exception of single-sided GC and AT skews at the telomeres (data not shown). Higher eukaryotes were not analyzed since the centromere region is typically unsequenced and replication timing data is unavailable. (B) *C. dubliniensis*- one additional non-centromeric skewed site was identified, but was not analyzed further. However, the skew levels at this site were weaker than all centromeric skew levels besides those of *CEN4*. Telomeres did not have skews as in *C. albicans* (not shown). (C) *L. elongisporus*- sequences were aligned at the GC skew zero-intersection point. *CEN6* is telomeric- the right side of the plot is the end of the chromosome. Skewed DNA on chromosomes 5 and 9 was flanked by long repetitive DNA (data not shown). Skew patterns were not identified in chromosomes 2 and 11, although a single long repetitive region was present in each of these chromosomes (not shown). The skew in chromosome 7 was different in that it was not associated with a high GC content. Chromosomes 2, 7 and 11 were not used for generating the average skew profile shown in Figure S8. (D) *Pichia stipitis*- clusters of repetitive same-sign overlapping GC and AT skews in very large intergenic regions (20–40 Kb), with overall high GC content (see also Figure S7) but no particular GC content extrema, were identified on eight out of nine chromosomes. Further investigation would be required in order to determine whether these sites correspond to centromeres and/or replication origins. (E) *C. lusitiniae*- sequences were aligned at the GC skew zero-intersection point. GC content (Figures S8, S9) dropped both locally, at the skew zero-intersection point, and globally, over a range of ∼30 Kb to each side. (F) *Y. lipolytica*- sequences were aligned at the AT skew zero-intersection point. The predicted sites based on skew patterns coincide or flank previously characterized centromere positions for chromosomes 1–5 [26]. The skew analysis also predicts the location of the chromosome 6 centromere, which has not been previously identified.
(0.44 MB PPT)

Table S1 Predicted centromere/origin locations by skew patterns in all species.
(0.06 MB DOC)
