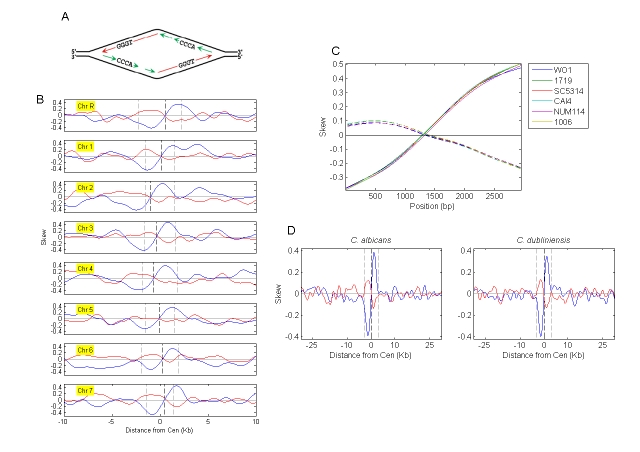# Correction: Epigenetically-Inherited Centromere and Neocentromere DNA Replicates Earliest in S-Phase

**DOI:** 10.1371/annotation/2aba8d24-7a24-4bbc-91f7-9b9e228cc84d

**Published:** 2011-04-28

**Authors:** Amnon Koren, Hung-Ji Tsai, Itay Tirosh, Laura S. Burrack, Naama Barkai, Judith Berman

Figure 3 is incorrect. Please see the correct version of Figure 3 here: 

**Figure:**