# Correction: Epigenetically-Inherited Centromere and Neocentromere DNA Replicates Earliest in S-Phase

**DOI:** 10.1371/annotation/d4e8b4eb-2385-4a46-938e-19bbae4fcf89

**Published:** 2011-04-28

**Authors:** Amnon Koren, Hung-Ji Tsai, Itay Tirosh, Laura S. Burrack, Naama Barkai, Judith Berman

Figure S6 is incorrect. Please see the correct version of Figure S6 here: 
