# Supplementary figures and images for: Epigenetically-Inherited Centromere and Neocentromere DNA Replicates Earliest in S-Phase
Source: PLoS Genet. 2010 Aug 19;6(8):e1001068. doi: 10.1371/journal.pgen.1001068 (PMC2924309; doi:10.1371/journal.pgen.1001068)

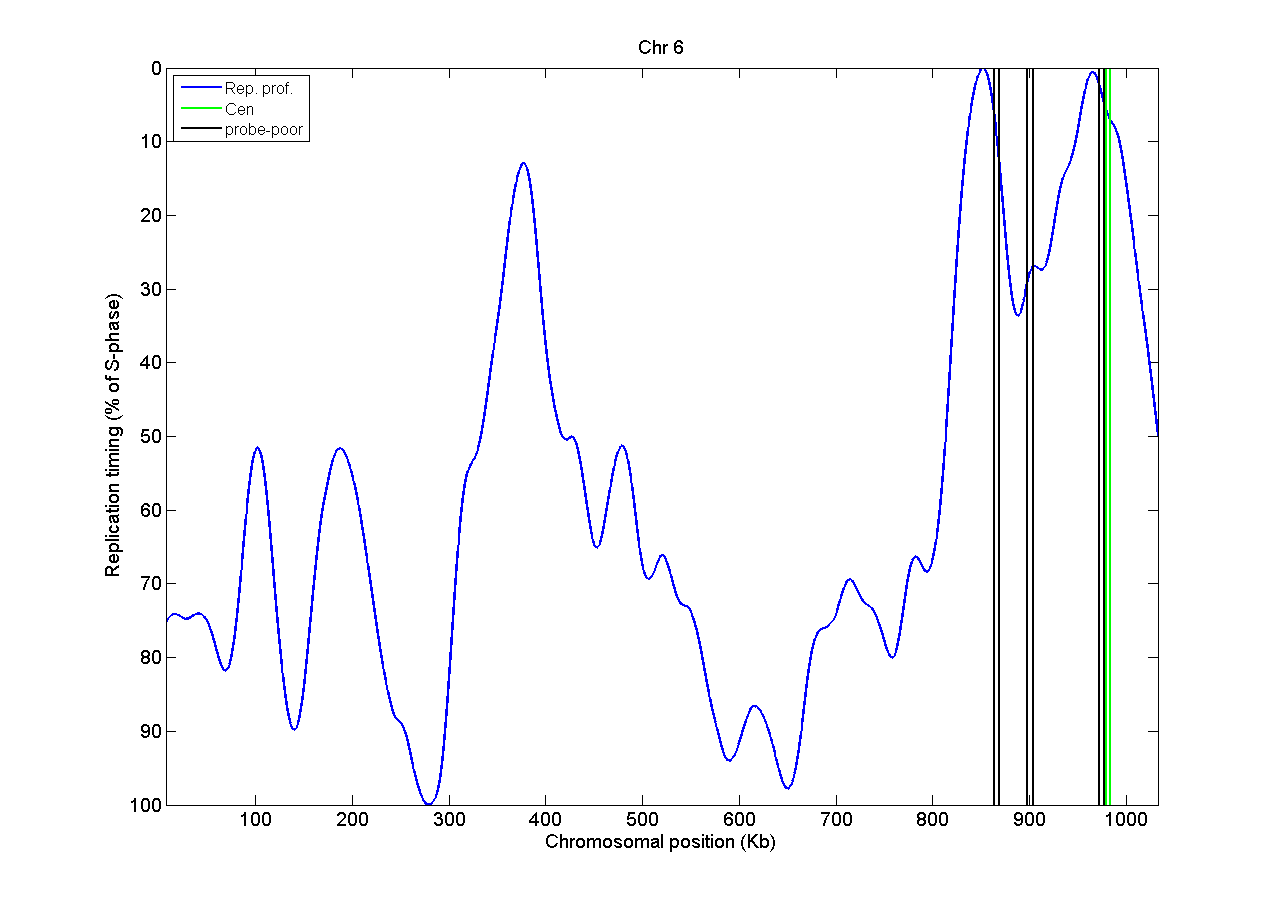

Supplement: Figure S1 — Replication profile of chromosome 6. As in Figure 1B. CEN6 replicated within the first 1% of S-phase with another origin on chromosome 6 replicating at 0%; however, in the vicinity of both origins there were large (>5 Kb) probe gaps; in addition, CEN6 is telocentric. For these reasons we consider the centromere replication timing for this chromosome as less reliable. Chromosome 6 was also not included in the C. albicans data used for generating Figure 1G and 1I. (0.14 MB TIF) [file pgen.1001068.s001.tif]

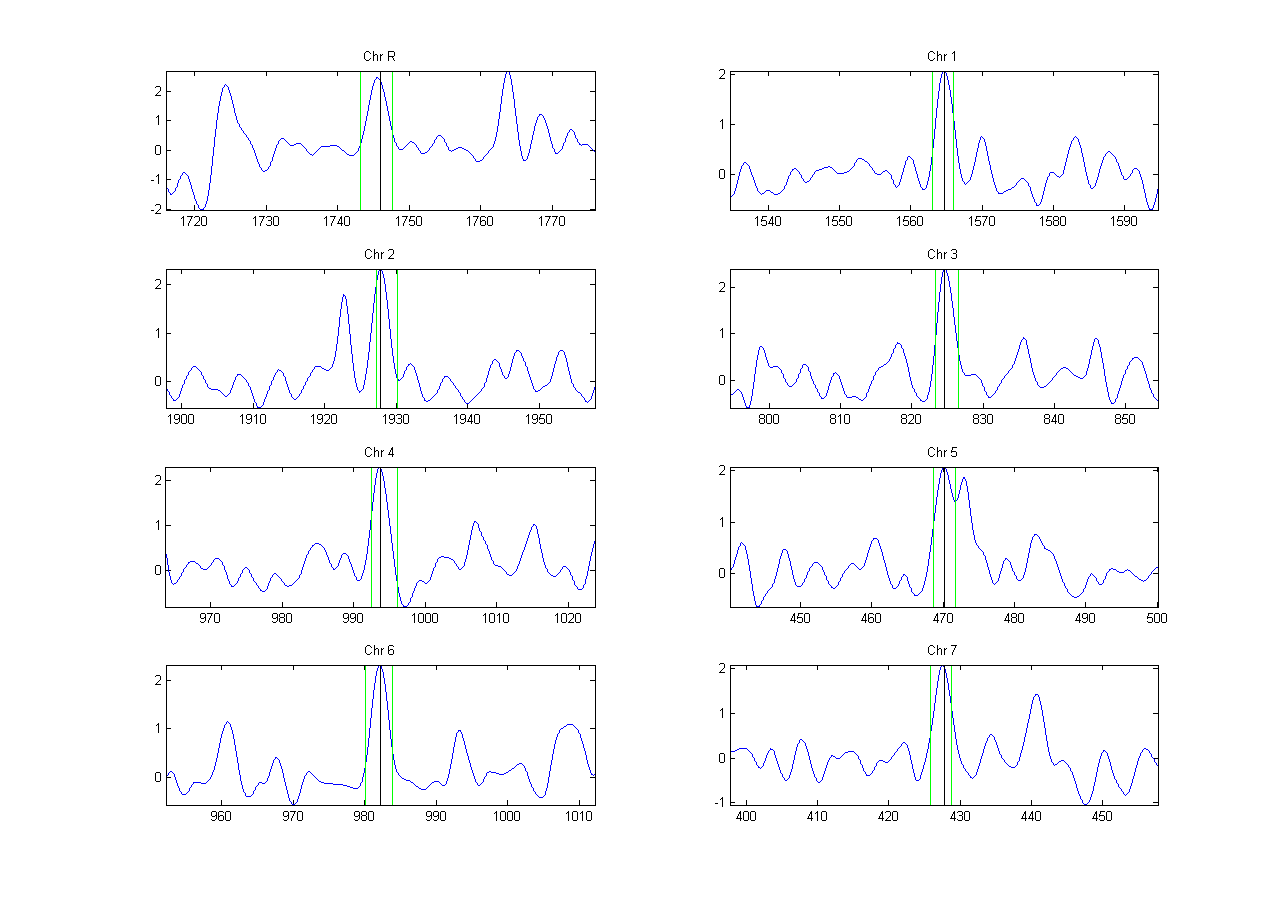

Supplement: Figure S2 — ChIP-chip data for all centromeric regions. The green lines correspond to the published centromere borders. The black line is the GC skew zero-intersection point (Figure 3B). x-axis: chromosomal position (Kb). y-axis: normalized log2 IP/WCE. (0.12 MB TIF) [file pgen.1001068.s002.tif]

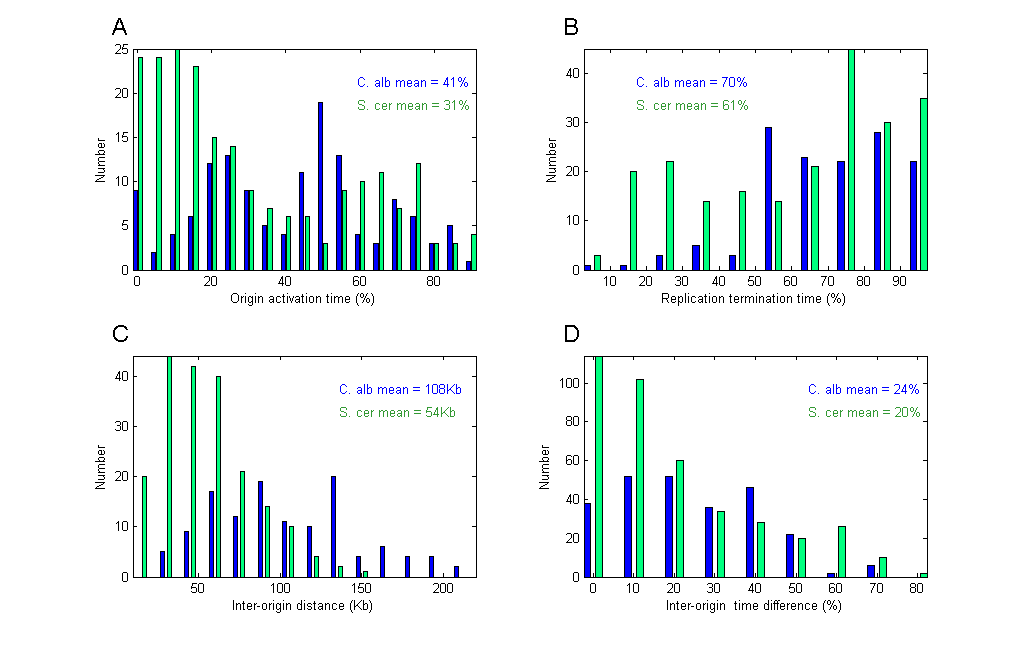

Supplement: Figure S3 — Properties of the replication program. Distribution of (A) origin initiation time, (B) replication termination time, (C) inter-origin distances, and (D) inter-origin time differences, for C. albicans (blue) and S. cerevisiae (green). Data from all the chromosomes was used to generate these figures. Origins numbers (and hence inter-origin distances) depend on smoothing parameters, and these were chosen to be maximally consistent between the two species (Methods). Our conclusion that the density of replication origins is significantly lower in C. albicans can also be reached independently by autocorrelation analysis, which is applied prior to smoothing (see [22]; data not shown). S. cerevisiae has more origins in early S-phase (A) which are clustered in space (C) and time (D) and therefore terminate early (B), thereby not contributing to the overall speed of S-phase. In C. albicans, this class of origins is not present, and origins are instead more uniformly distributed in S-phase (A), are more separated in space (C) and time (D) and are associated with replication forks that extend over a larger portion of S-phase, thus terminating late (B). The percentage of C. albicans cells in G1, S and G2 phases (Figure 1A) was 47%, 24% and 29%, respectively. This corresponds to 30.5, 15.6 and 18.9 minutes, for a total generation time of 65 minutes. In comparison, S-phase in S. cerevisiae lasts 17 minutes [22]. Thus, while length of S-phase is not extended in C. albicans relative to S. cerevisiae, in C. albicans there is less origin redundancy. (0.19 MB TIF) [file pgen.1001068.s003.tif]

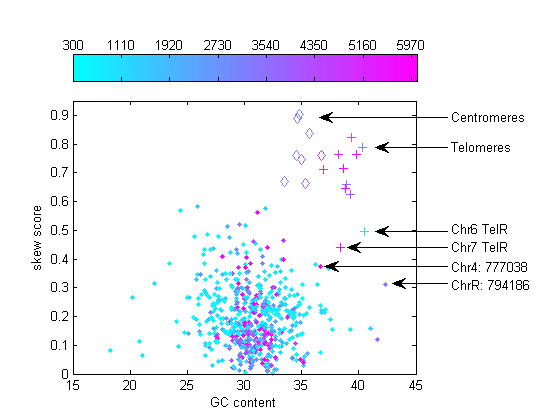

Supplement: Figure S4 — Identification of skew locations. Since GC skew is also associated with high GC content, we used both criteria in order to scan for skew patterns. In the figure, each dot represents an intergenic region. Centromeres are represented by open diamonds and telomeres by ‘+’ signs. The Y-axis refers to the summed skew level for GC skews that change from negative to positive (the highest score was used for intergenic regions which had more than one such skew pattern). The colorcode denotes the length of the intergenic region. Besides the eight centromeres, nine telomeric sites could be identified with comparable levels of GC skew and even higher GC content (and see Figure S5). GC skews at telomeres have also been described in S. cereviaise [Gierlik et al], however they are single sided rather than asymmetrical (data not shown), probably arising due to the uni-directional replication from the terminal origins on each chromosome to the ends of that chromosome. Two additional telomeric sites (one of them, Tel6R, in a short intergenic region- see Figure S5) and two internal replication origins could be identified with weaker skew levels. The non-centromeric origins associated with skew signals were not early replicating compared to average. Other than those shown in the figure, replication origins in general did not have stronger skew levels than non-origin sites (not shown). The high correlation between strong skew patterns and elevated GC content (see also Figures S6, S7) suggests that the same mutational mechanism may cause both. Centromere and telomere loci could also be identified with other skew parameters, such as a minimal GC skew level on either side of a zero-intersection point associated with a reverse-sign AT skew. [Gierlik A, Kowalczuk M, Mackiewicz P, Dudek MR, Cebrat S (2000) Is there replication-associated mutational pressure in the Saccharomyces cerevisiae genome? J Theor Biol 202: 305–314.] (0.07 MB TIF) [file pgen.1001068.s004.tif]

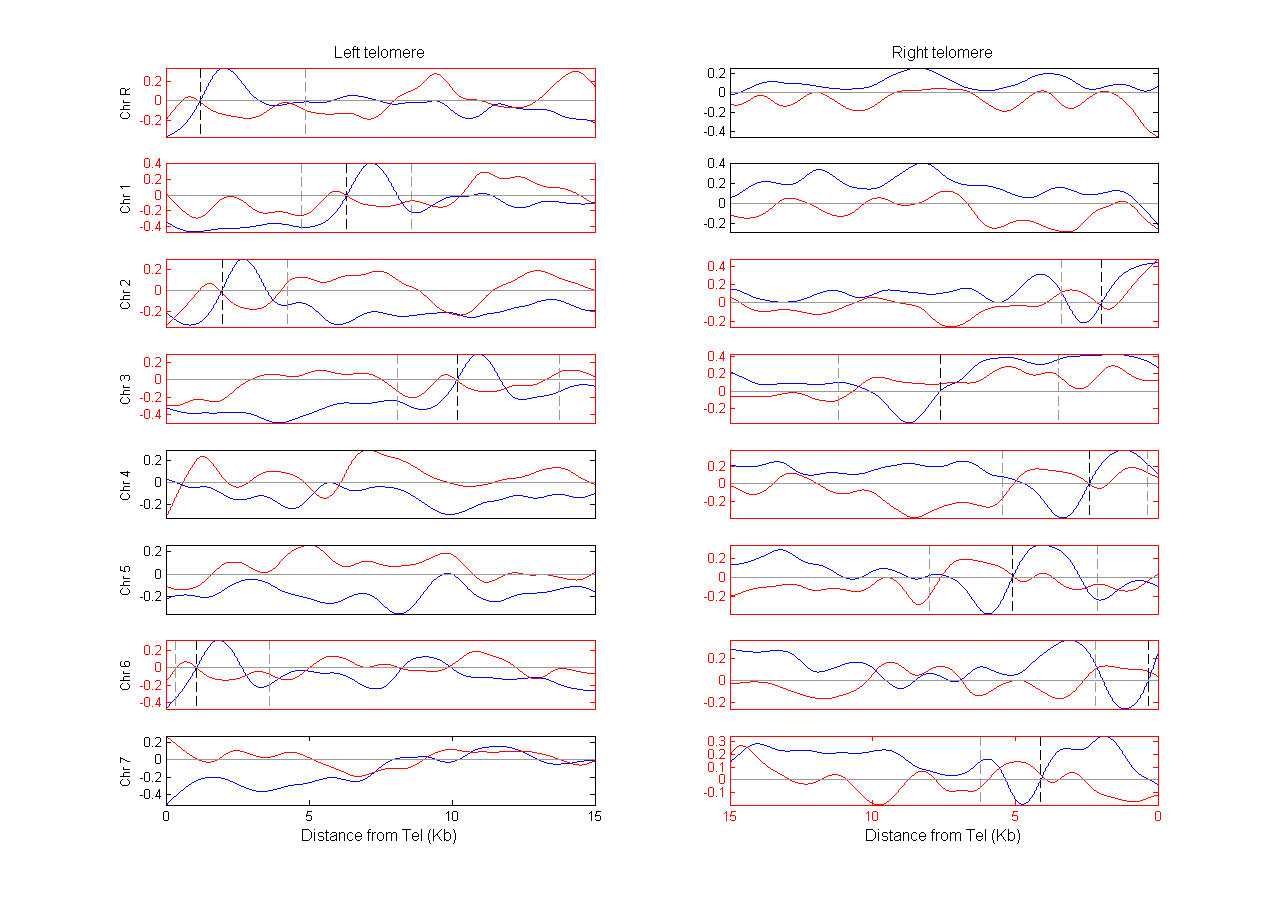

Supplement: Figure S5 — Telomere skew patterns. As in Figure 3A; left and right C. albicans telomeres are shown on the left and right subplot columns, respectively. Dashed grey lines- borders of the intergenic region containing the skew pattern; when one dashed gray line is present, the other is the end of the chromosome. Red boxes denote telomeres identified by the genomic search for GC skew (Figure S4). The DNA region from the most terminal replication origin site to the corresponding end of the chromosome is always replicated in one direction and will thus show skew patterns, depending on the presence of mutational strand biases in the studied organism, and the absence of selective pressure in this region. For instance, GC and AT skews are observed at S. cerevisiae telomeres albeit they are not asymmetrical but rather single-sided (data not shown). This is consistent with multiple potential origins sites at telomeres [33] that are active alternatively. The identification of asymmetrical skew patterns at C. albicans telomeres suggests that they contain a single, isolated, active replication origin. This is also consistent with the differences in telomere replication timing between the two yeasts (Figure 1H). (0.15 MB TIF) [file pgen.1001068.s005.tif]

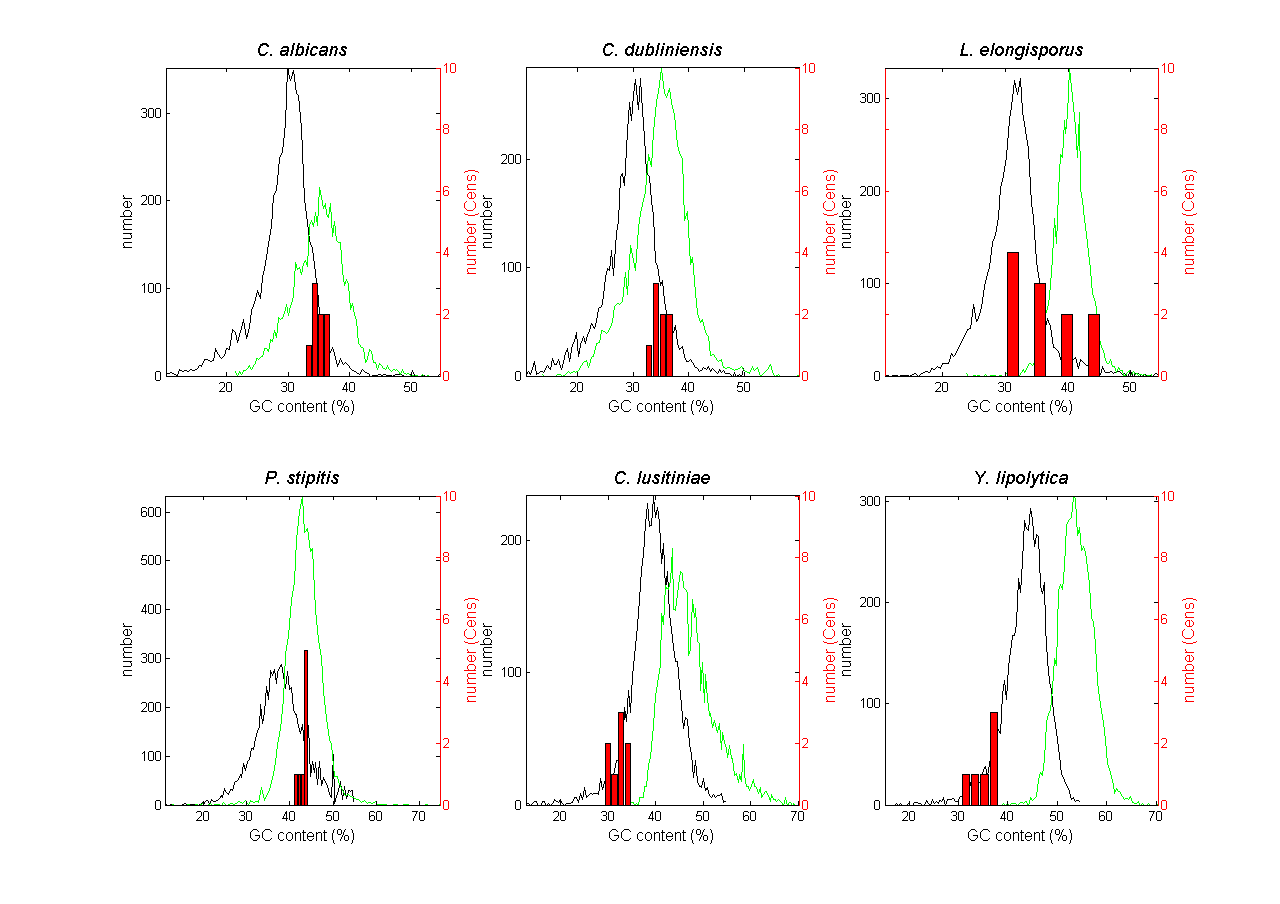

Supplement: Figure S7 — GC content of centromeres. Shown are the distributions of GC content for ORFs (green), intergenic regions (black) and centromeres (red). (0.16 MB TIF) [file pgen.1001068.s007.tif]

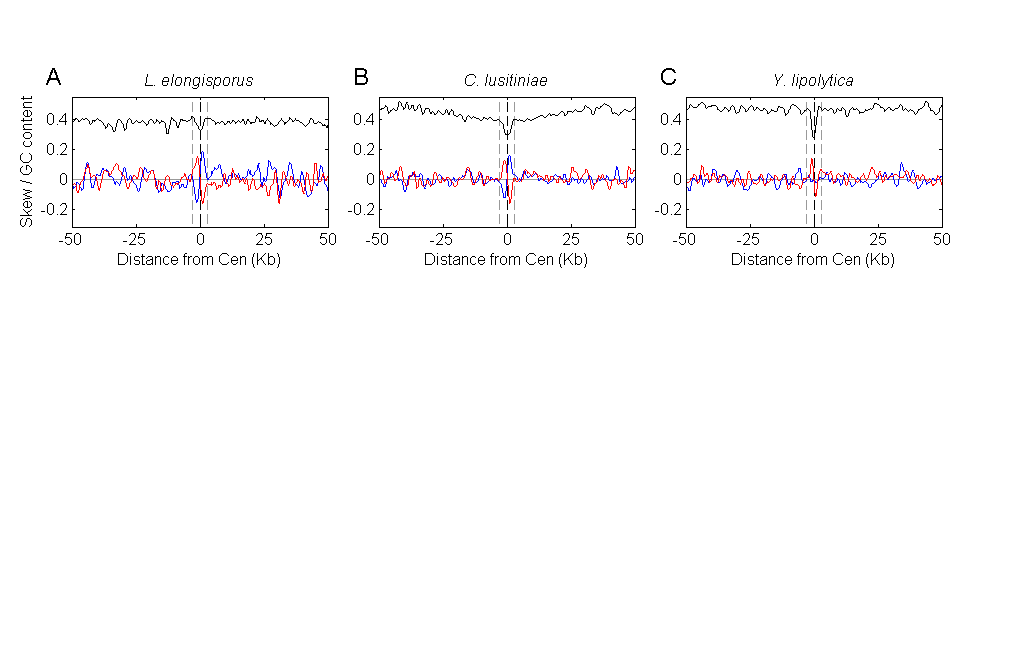

Supplement: Figure S8 — Since skew patterns provide a sequence footprint for ancient centromere-origins, we asked if similar patterns could be identified over a broader phylogenetic range by searching several yeast genomes for intergenic regions with distinctive skew and GC content values (Methods and see Figure S4). The figure shows the average skew and GC content patterns, with individual chromosomes shown in Figure S9. Skew and GC content (black) are shown on the same scale, as level or fraction, respectively. We identified skew patterns, which appeared only once per chromosome, in L. elongisporus (A) and C. lusitinae (B), as well as in the distantly related species Yarrowia lipolytica (C). In Y. lipolytica, our approach re-identified the five known centromere positions in this organism [26]. Remarkably, the latter were identified by searching for chromosomal sequences that function as DNA replication origins [27] and subsequent work showed that, for Y. lipolytica plasmids, replication origin and centromere activity are inter-dependent [26]. Interestingly, an AT skew was present to similar extents in the three species as it was in C. albicans and C. dubliniensis (Figure 3); in contrast, GC skew followed an evolutionary path from being not present (in Y. lipolytica), to equivalent in magnitude to the AT skew (in the predicted centromeres), and to significantly stronger (in C. albicans and C. dubliniensis). GC content was correlated with the extent of GC skew: it showed a local drop when a GC skew was absent, but was progressively higher with the increase in GC skew levels (see also Figure S7). Together, this suggests that different replication-dependent mutational mechanisms operated in different lineages. (0.06 MB TIF) [file pgen.1001068.s008.tif]

## Slide 1
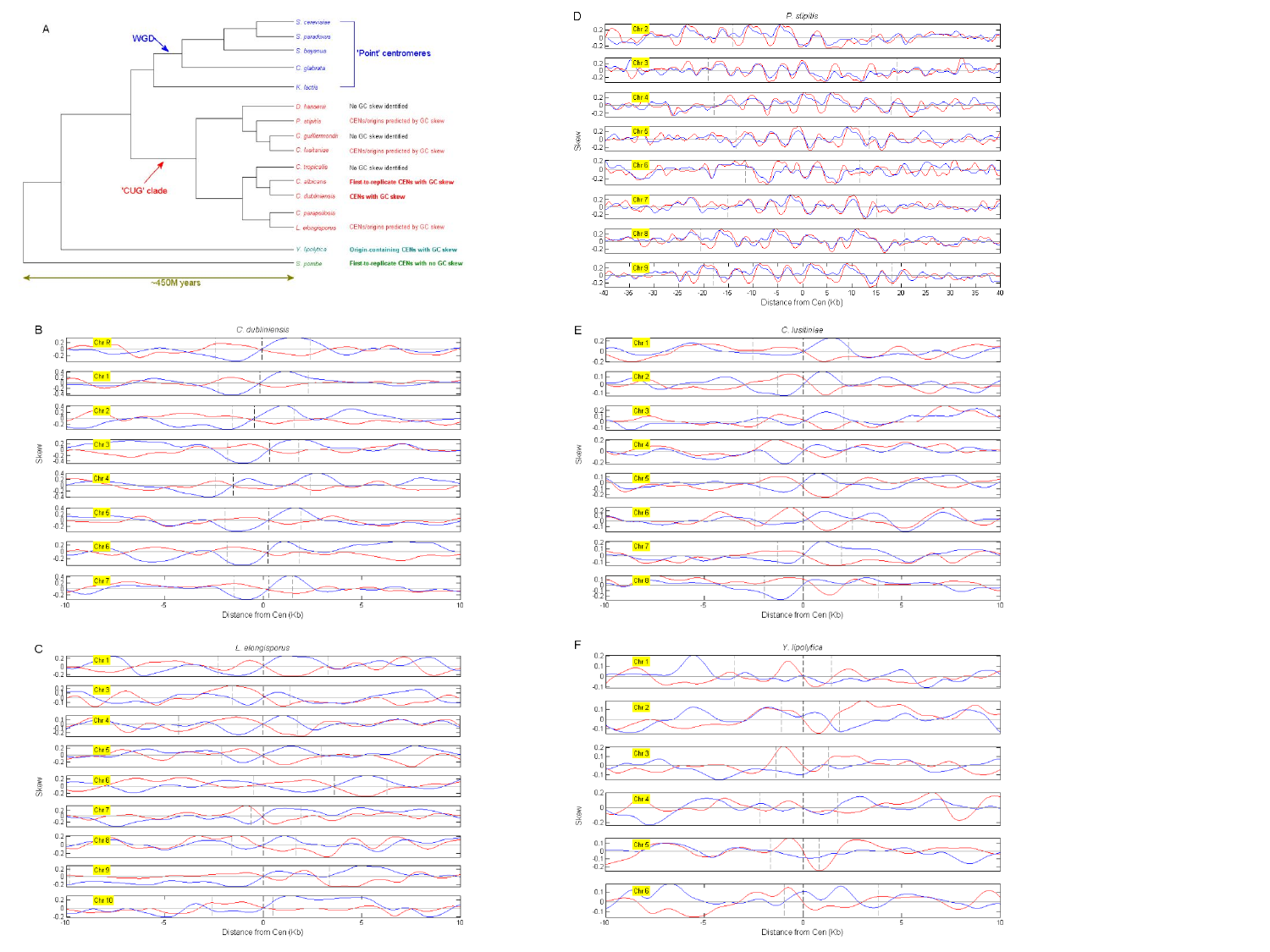

Supplement: Figure S9 — Skew patterns for different species. (A) Phylogenetic tree of species analyzed in this study and related species (modified from Genolevures (http://wwwgenolevuresorg/indexhtml)). WGD: whole genome duplication. ‘CUG’ clade: the Candida species clade, in which the CUG codon encodes Serine instead of Threonine. ‘Point centromeres’: centromeres defined by a short consensus sequence. (B–F) As in Figure 3B; GC and AT skews for separate chromosomes in the indicated species. Grey- borders of the intergene containing the skew pattern, except for C. dubliniensis- borders of reported Cse4 binding sites. See Table S1 for coordinates of skew zero-intersection points and corresponding intergenic region borders. No skew signals were identified in C. guillermondii, D. hansenii and C. tropicalis. However, since centromere locations are unknown for these species, it remains possible that centromeres are associated with skew and GC content patterns that are not strong enough to be recognized unequivocally. No skew signals were identified in the S. pombe genome despite the presence of active (and early) replication origins within the centromere regions [28]; this could be explained by alternate firing of different origins within the centromere region, by a different mutational mechanisms, or by functional constraints. No skew signals were identified in the S. cerevisiae genome, with the exception of single-sided GC and AT skews at the telomeres (data not shown). Higher eukaryotes were not analyzed since the centromere region is typically unsequenced and replication timing data is unavailable. (B) C. dubliniensis- one additional non-centromeric skewed site was identified, but was not analyzed further. However, the skew levels at this site were weaker than all centromeric skew levels besides those of CEN4. Telomeres did not have skews as in C. albicans (not shown). (C) L. elongisporus- sequences were aligned at the GC skew zero-intersection point. CEN6 is telomeric- the right side of the [file pgen.1001068.s009.ppt]
