# Supplementary material for: Epigenetically-Inherited Centromere and Neocentromere DNA Replicates Earliest in S-Phase
Source: PLoS Genet. 2010 Aug 19;6(8):e1001068. doi: 10.1371/journal.pgen.1001068 (PMC2924309; doi:10.1371/journal.pgen.1001068)
Supplement: Table S1 — Predicted centromere/origin locations by skew patterns in all species. (0.06 MB DOC) [file pgen.1001068.s010.doc]

Table S1. Predicted centromere/origin locations by skew patterns in all species

C. albicans

| Chr | Intergenic region- left border | Intergenic region- right border | GC skew zero-intersection point |
| --- | --- | --- | --- |
| R | 1743293 | 1748790 | 1746006 |
| 1 | 1561482 | 1578966 | 1564658 |
| 2 | 1923104 | 1930309 | 1927785 |
| 3 | 822375 | 826160 | 824518 |
| 4 | 990509 | 996164 | 993772 |
| 5 | 467791 | 473410 | 470131 |
| 6 | 978543 | 984556 | 982170 |
| 7 | 423406 | 429946 | 427735 |

*C. dubliniensis*

| Chr | Intergenic region- left border | Intergenic region- right border | GC skew zero-intersection point |
| --- | --- | --- | --- |
| R | 1713450 | 1722609 | 1718493 |
| 1 | 1594163 | 1611889 | 1598250 |
| 2 | 1941422 | 1947217 | 1944848 |
| 3 | 869105 | 869802 | 869388 |
| 4 | 1028924 | 1036395 | 1030719 |
| 5 | 494073 | 500591 | 497510 |
| 6 | 1001458 | 1009565 | 1005103 |
| 7 | 434210 | 439177 | 437015 |

*L. elongisporus*

| Chr | Intergenic region- left border | Intergenic region- right border | GC skew zero-intersection point |
| --- | --- | --- | --- |
| 1 | 1451774 | 1457402 | 1454081 |
| 2 | Not identified | Not identified | Not identified |
| 3 | 801410 | 804331 | 802976 |
| 4 | 50972 | 57004 | 55269 |
| 5 | 740603 | 745641 | 742698 |
| 6 | 1241160 | 1246124 | 1244175 |
| 7 | 892974 | 895527 | 893593 |
| 8 | 18056 | 21336 | 19666 |
| 9 | 418797 | 431998 | 428643 |
| 10 | 447460 | 450525 | 450030 |
| 11 | Not identified | Not identified | Not identified |

*P. stipitis*

| Chr | Intergenic region- left border | Intergenic region- right border |
| --- | --- | --- |
| 1 | Not identified | Not identified |
| 2 | 97050 | 125137 |
| 3 | 1668430 | 1706643 |
| 4 | 1414539 | 1450323 |
| 5 | 1030956 | 1057832 |
| 6 | 646811 | 669916 |
| 7 | 886381 | 916530 |
| 8 | 258136 | 299533 |
| 9 | 290537 | 326613 |

*C. lusitiniae*

| Chr | Intergenic region- left border | Intergenic region- right border | GC skew zero-intersection point |
| --- | --- | --- | --- |
| 1 | 1055556 | 1060408 | 1058107 |
| 2 | 1806190 | 1809472 | 1807490 |
| 3 | 1158004 | 1162378 | 1160319 |
| 4 | 140508 | 145188 | 142975 |
| 5 | 286972 | 290895 | 289157 |
| 6 | 278061 | 282979 | 280484 |
| 7 | 375217 | 378464 | 376511 |
| 8 | 158650 | 164422 | 160603 |

*Y. lipolytica*

| Chr | Intergenic region- left border | Intergenic region- right border | AT skew zero-intersection point |
| --- | --- | --- | --- |
| 1 | 1004263 | 1009200 | 1007740 |
| 2 | 714401 | 717361 | 715506 |
| 3 | 2136332 | 2138988 | 2137702 |
| 4 | 1924814 | 1928725 | 1926985 |
| 5 | 1835944 | 1838411 | 1837584 |
| 6 | 1944840 | 1949620 | 1945785 |
