# Supplementary figures and images for: Correction: Epigenetically-Inherited Centromere and Neocentromere DNA Replicates Earliest in S-Phase
Source: PLoS Genet. 2011 Apr 28;7(4):10.1371/annotation/d4e8b4eb-2385-4a46-938e-19bbae4fcf89. doi: 10.1371/annotation/d4e8b4eb-2385-4a46-938e-19bbae4fcf89 (PMC3084637; doi:10.1371/annotation/d4e8b4eb-2385-4a46-938e-19bbae4fcf89)

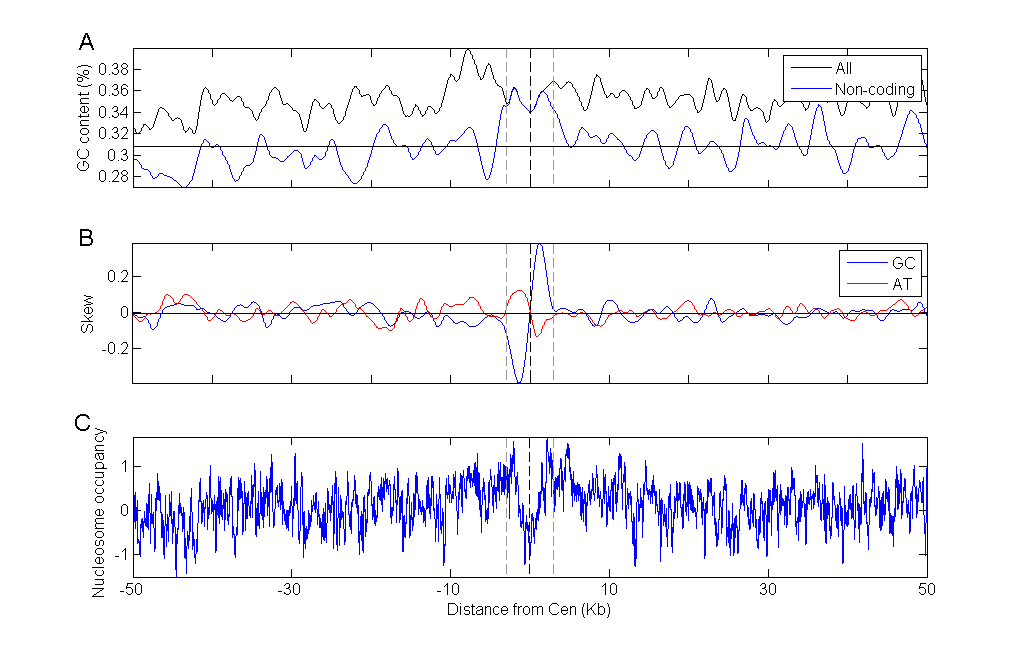

Supplement: Supplementary file 1 [file pgen.d4e8b4eb-2385-4a46-938e-19bbae4fcf89.s001.tif]
